# Risk of neonatal SARS-CoV-2 infection: a retrospective cohort study based on infected mothers with gestational diabetes mellitus

**DOI:** 10.3389/fendo.2025.1483962

**Published:** 2025-01-30

**Authors:** Jing Ni, Yongfei Zheng, Jiaqi Tian, Lin Zhang, Shuyin Duan

**Affiliations:** ^1^ School of Public Health, Shandong First Medical University & Shandong Academy of Medical Sciences, Jinan, China; ^2^ Clinical Medical Research Center for Women and Children Diseases, Key Laboratory of Birth Regulation and Control Technology of National Health Commission of China, Shandong Provincial Maternal and Child Health Care Hospital Affiliated to Qingdao University, Jinan, China; ^3^ Shandong Provincial Key Medical and Health Laboratory of Women’s Occupational Exposure and Fertility Preservation, Jinan, China; ^4^ Jinan (Preparatory) Key Laboratory of Women’s Diseases and Fertility Preservation, Jinan, China

**Keywords:** COVID-19 pandemic, infection, pregnancy, gestational diabetes mellitus, neonatal susceptibility

## Abstract

**Background:**

The COVID-19 pandemic has posed unprecedented challenges to global public health, especially for pregnant women and their offspring. However, little is known about the impact of maternal SARS-CoV-2 infection on neonatal outcomes, particularly in the context of coexisting gestational diabetes mellitus (GDM).

**Methods:**

Hospitalized pregnant women with SARS-CoV-2 infection were retrospectively enrolled between November 2022 and January 2023, and matched with pregnant subjects free of SARS-CoV-2 infection based on their propensity scores. All women were tested for SARS-CoV-2 upon admission as part of routine procedures, then divided into groups of pregnant women with SARS-CoV-2 infection and GDM (SARS2+GDM), pregnant women with SARS-CoV-2 infection but without GDM (SARS2+noGDM), and pregnant women without SARS-CoV-2 infection or GDM (Normal group). A logistic regression model was used to study the risk of GDM, perinatal SARS-CoV-2 infection, and their interaction on neonatal SARS-CoV-2 infection.

**Results:**

Of 378 pregnant women with SARS-CoV-2 infection, the neonatal infection rate was higher in the GDM group as compared to the SARS-CoV-2 infection only group, but both SARS-CoV-2 infection rates were lower than that of the normal control group. Logistic regression analysis identified an interaction between maternal SARS-CoV-2 infection and GDM on neonatal infection, where maternal SARS-CoV-2 infection (odds ratio [OR] = 0.31, 95%CI: 0.22-0.44) and vaccination for anti-SARS-CoV-2 (OR = 0.70, 95%CI: 0.50-0.98) were associated with lower odds of neonatal infection, while higher pre-pregnancy body mass index (BMI) (OR = 1.06, 95% CI: 1.02-1.10) and GDM (OR = 1.97, 95%CI: 1.21-3.21) were associated with higher odds of neonatal infection.

**Conclusions:**

We demonstrate that the coexistence of GDM and perinatal SARS-CoV-2 infection was associated with an increased probability of neonatal SARS-CoV-2 infection.

## Background

COVID-19 is an infectious disease caused by the severe acute respiratory syndrome coronavirus 2 (SARS-CoV-2), emerged in December 2019 with a very high transmissibility rate and has had numerous negative impacts on global life (1). On May 5, 2023, the World Health Organization (WHO) declares end to COVID-19 as a global health emergency, but the emergence of SARS-CoV-2 variants and SARS-CoV-2 reinfection raise additional concerns (2, 3). To date, COVID-19 has spread rapidly across the world, affecting more than 200 million people and causing over 6 million deaths as of June 2023 (4). In terms of clinical manifestations, COVID-19 causes a range of symptoms, from mild to severe, including fever, cough, shortness of breath, and loss of taste or smell. In some cases, it leads to organ failure or death (5–7). While most individuals recover within a few weeks, some may experience prolonged health issues after contracting the virus.

Among the most vulnerable populations are pregnant women and their newborns, who may face increased risks of complications and adverse outcomes (8). Literature on the outcomes of SARS-CoV-2 infections during pregnancy is slowly building up. Available studies indicate that maternal infections with SARS-CoV-2 during pregnancy usually do not have severe consequences for mother and child. However, some pregnant women become seriously ill with COVID-19, and the primary risk to the infant appears to arise from maternal illness (9). Data shows that even when the fetus is not infected, maternal SARS-CoV-2 infection can indirectly prime the fetal immune response (10). For instance, a study conducted during the COVID-19 pandemic in China found that the majority of mothers were infected with SARS-CoV-2 during the third trimester, and as a result, their offspring exhibited reduced motor, communication, and social performance at 3 months of age compared to normal levels (11).

Gestational diabetes mellitus (GDM) is the most common complication during pregnancy and is defined as any degree of glucose intolerance with onset or first recognition during pregnancy (12). One of the risk factors that may affect the severity and prognosis of COVID-19 in pregnancy is abnormal blood glucose level, which is affected by pre-existing diabetes or GDM (13–15). The association of GDM with SARS-CoV-2 infection in pregnant women is complex and multifaceted. On one hand, GDM may increase the risk of SARS-CoV-2 infection and severity in pregnant women due to impaired immune function, increased inflammation, and endothelial dysfunction (13). On the other hand, SARS-CoV-2 infection may causes viral-induced stress, cytokine storm, and reduced access to health care, which worsen blood glucose control and increase the risk of developing or exacerbating diabetes and GDM in pregnant women (16, 17). The effects of GDM and SARS-CoV-2 infection on offspring health may be significant and long-lasting, although some studies have reported no significant impact. Regarding offspring, they are speculated to affect fetal growth and development, resulting in intrauterine growth restriction, macrosomia, congenital anomalies, or stillbirth (18). For the mother, these conditions may increase the risk of adverse pregnancy outcomes, such as preterm birth, preeclampsia, cesarean delivery, and postpartum hemorrhage (19). Also, some studies with limited evidence suggest that SARS-CoV-2 infection may reprogram the offspring’s metabolic and immune systems for life-long susceptibility to obesity, diabetes, and infectious diseases (20).

While some studies suggest the possibility of vertical transmission, definitive proof of SARS-CoV-2 crossing the placenta remains limited. For instance, Dustin et al. provided evidence of transplacental transfer of SARS-CoV-2-specific IgG antibodies in pregnant women, highlighting the potential for maternal immune protection to extend to the fetus (21). Particularly, Alexandre et al. documented a case of vertical transmission of SARS-CoV-2 in a neonate whose mother was infected during the third trimester, with the infant exhibiting neurological complications postnatally (22). In the context to GDM, its interaction with maternal SARS-CoV-2 infection may further complicate neonatal outcomes. As being widely known, GDM induces chronic inflammation and alter placental function, which could exacerbate the risks associated with viral infections during pregnancy. Therefore, this study was conducted to investigate whether the coexistence of GDM and SARS-CoV-2 infection increases the likelihood of neonatal infection, building on the hypothesis that GDM might amplify the inflammatory response and placental dysfunction, thereby increasing the risk of vertical transmission or adverse neonatal outcomes.

## Methods

### Patients and clinical data

This study is a part of our registered birth cohort entitled *Maternal SARS-CoV-2 Infection and Offspring Susceptibility* (MSIOS, clinical registration number: MR-37-23-019409). The MSIOS cohort is a single-center retrospective study with pregnant women who gave birth between November 3, 2022 and January 6, 2023, one month before and after the easing of COVID-19 control measures in China (December 7, 2022). All women included in this study were hospitalized for childbirth, during which SARS-CoV-2 testing was performed as part of routine admission procedures regardless of symptoms. In parallel, women hospitalized primarily for COVID-19 treatment were excluded. We categorized the women into three main groups: a) Women with GDM and SARS-CoV-2 infection (SARS2+GDM), b) Women without GDM but with SARS-CoV-2 infection (SARS2+noGDM), and c) Women without GDM and SARS-CoV-2 infection (Normal group). Clinical information, including demographic characteristics, medication, and laboratory examination results, were collected by reviewing medical records. Additional information, such as COVID-19 symptoms in pregnant women, paternal baseline characteristics, and neonatal SARS-CoV-2 infection status, was obtained by following up the patients within one month after discharge. This study was approved by the Institutional Review Board of the Shandong Provincial Maternal and Child Health Care Hospital Affiliated to Qingdao University. The personal information of all study participants was anonymized prior to statistical analysis.

### Definitions

According to the American Diabetes Association (23), GDM is defined as diabetes diagnosed in the second or third trimester of pregnancy that has not been clearly overt diabetes prior to gestation. In this study, GDM was diagnosed by referring to the criteria recommended by the WHO in 2013 (24): fasting plasma glucose  ≥ 5.1 mmol/L, and/or 75 g oral glucose tolerance test for 1-h plasma glucose  ≥ 10.0 mmol/L, and/or 2-h plasma glucose ≥ 8.6 mmol/L. SARS-CoV-2 infection was determined by nucleic acid test, irrespective of the presence or absence of clinical symptoms such as fever, cough, shortness of breath or difficulty breathing, fatigue, muscle or body aches, headache, new loss of taste or smell, and sore throat.

### Covariates and primary outcomes

From three aspects of maternal, paternal, and neonatal, the covariates were included from variables that may influence the maternal and fetal health. The maternal factors were age, pre-pregnancy BMI, education level, SARS-CoV-2 vaccination, folic acid supplementation, medication during pregnancy, age at first pregnancy and menarche, gravidity, preterm birth, abortion, living birth, menstrual cycle regularity, gestational age at delivery, delivery mode, and conception method. Paternal factors such as age, BMI, cigarette and alcohol consumption, and education background were also investigated. The sex of the newborn was examined as a neonatal factor. Neonatal infection of SARS-CoV-2 was the primary outcome of the study.

### Statistical analysis

All data extracted from the MSIOS cohort was collected using Microsoft Excel 2019. The data visualization and statistical analysis were performed using R version 4.3.0 in RStudio 2023.06.0 + 421 for Windows. Baseline characteristics were described as mean ± standard deviation for continuous variables and frequency with proportion for categorical variables. A logistic regression model was established to examine the associations between GDM, SARS-CoV-2 infection, and neonatal SARS-CoV-2 infection. A p-value less than 0.05 was considered statistically significant unless otherwise indicated.

## Results

### Demographic characteristics of study subjects

**Figure 1:**
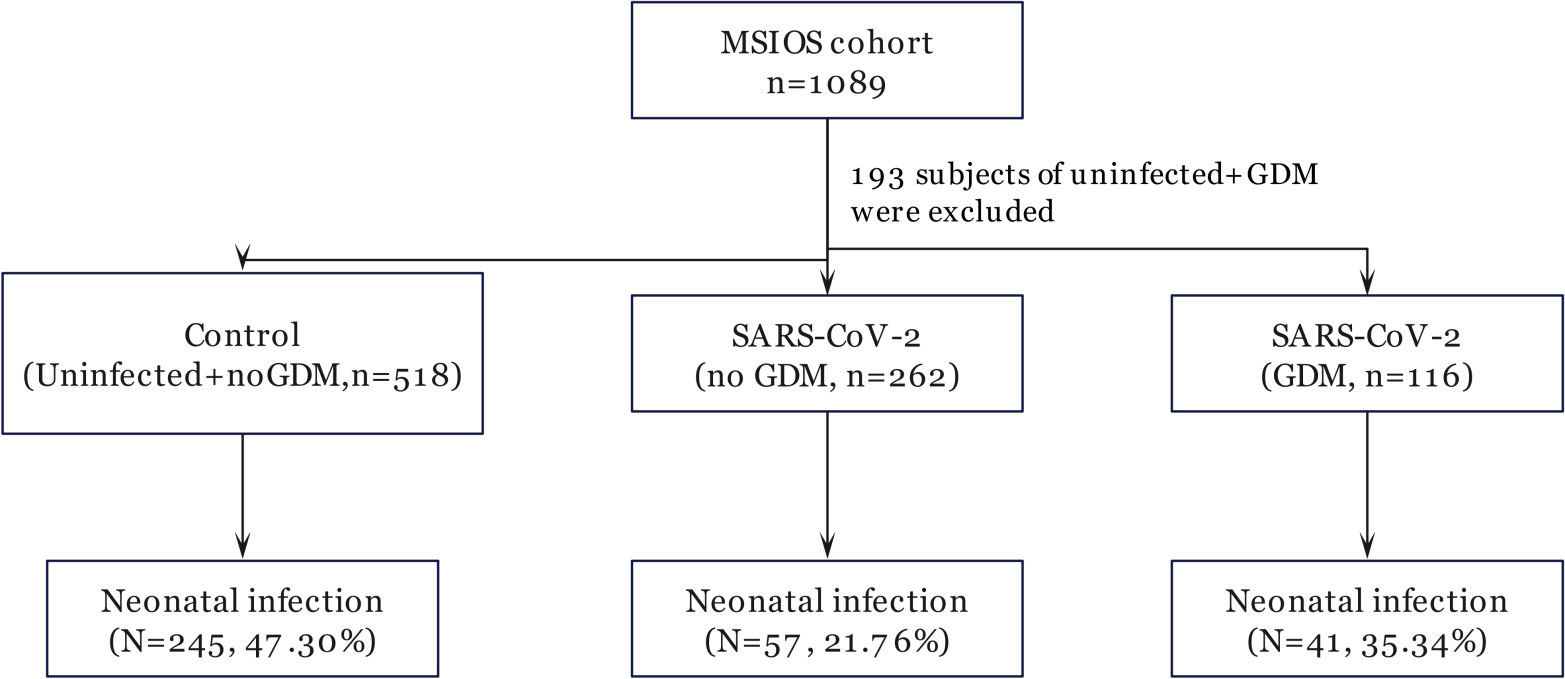
Flow chart of participant selection.

A total of 896 pregnant women were enrolled from the MSIOS cohort (**Figure 1**, **Table 1**). Of these, 518 subjects were healthy pregnant women free of GDM and SARS-CoV-2 infection, treated as normal control; while 378 subjects were diagnosed with SARS-CoV-2 infection, and 116 of 378 subjects were complicated with GDM, and 262 were only SARS-CoV-2 infection cases.

**Table 1:** Baseline characteristics of pregnant women and comparison between SARS-CoV-2 infected and uninfected subjects.

Variables	Uninfected (*N=518*)	Infected (*N=378*)	*p* value
Maternal factors			
Age at pregnancy (yrs)	30.30 ± 4.02	30.99 ± 4.23	0.014
Pre-pregnancy BMI (kg/m^2^)	26.31 ± 4.10	27.15 ± 3.90	0.002
Education background (n)			0.490
Uneducated/Primary school graduate	23 (4.44%)	12 (3.17%)	
High school graduate	43 (8.30%)	24 (6.35%)	
University Graduate	380 (73.36%)	291 (76.98%)	
Postgraduate	72 (13.90%)	51 (13.49%)	
Vaccination for anti-SARS-CoV-2 (n)			0.006
No	130 (25.10%)	65 (17.20%)	
Yes	388 (74.90%)	313 (82.80%)	
Folic acid supplementation (n)			0.001
No	20 (3.86%)	35 (9.26%)	
Yes	498 (96.14%)	343 (90.74%)	
Medication (n)^∫^			0.201
No	476 (91.89%)	337 (89.15%)	
Yes	42 (8.11%)	41 (10.85%)	
Age at first pregnancy (yrs)	27.44 ± 3.72	27.35 ± 3.39	0.692
Gravidity (n)			0.924
0	207 (39.96%)	149 (39.42%)	
≥1	311 (60.04%)	229 (60.58%)	
Preterm birth (n)			0.099
0	512 (98.84%)	367 (97.09%)	
≥1	6 (1.16%)	11 (2.91%)	
Abortion (n)			0.959
0	489 (94.40%)	358 (94.71%)	
≥1	29 (5.60%)	20 (5.29%)	
Age at menarche (yrs)	14.08 ± 2.07	13.69 ± 0.84	<0.001
Menstrual days (days)	5.83 ± 0.85	5.79 ± 0.80	0.450
Menstrual Cycle regularity (n)			0.231
Regular	479 (92.47%)	358 (94.71%)	
Irregular	39 (7.53%)	20 (5.29%)	
Gestational age at delivery (wks)	39.02 ± 2.77	39.16 ± 1.31	0.329
Delivery mode (n)			0.248
Vaginal delivery	313 (60.42%)	213 (56.35%)	
Cesarean delivery	205 (39.58%)	165 (43.65%)	
Conception method (n)			0.667
Artificial technology	23 (4.44%)	20 (5.29%)	
Natural pregnancy	495 (95.56%)	358 (94.71%)	
Paternal factors			
Age	30.81 ± 5.50	31.41 ± 5.84	0.115
BMI	25.20 ± 3.43	25.32 ± 4.11	0.641
Smoke (n)			0.476
No	370 (71.43%)	279 (73.81%)	
Yes	148 (28.57%)	99 (26.19%)	
Alcohol consumption (n)			0.986
No	329 (63.51%)	239 (63.23%)	
Yes	189 (36.49%)	139 (36.77%)	
Education background (n)			0.568
Uneducated/Primary school graduate	57 (11.00%)	38 (10.05%)	
High school graduate	44 (8.49%)	25 (6.61%)	
University Graduate	343 (66.22%)	266 (70.37%)	
Postgraduate	74 (14.29%)	49 (12.96%)	
Neonatal factors			
SARS-CoV-2 infection (n)			<0.001
Uninfected	273 (52.70%)	280 (74.07%)	
Infected	245 (47.30%)	98 (25.93%)	
Sex of the newborn (n)			0.896
Male	261 (50.39%)	193 (51.06%)	
Female	257 (49.61%)	185 (48.94%)	

^∫^any drugs taken during pregnancy.

GDM, gestational diabetes mellitus; BMI, body mass index.

Compared with the normal control group, subjects in the SARS-CoV-2 infection group had higher age at pregnancy and pre-pregnancy BMI, while lower rate of neonatal SARS-CoV-2 infection. There were no statistically significant differences in the rest maternal, paternal, and neonatal factors.

### Effect of GDM on neonatal SARS-CoV-2 infection

To investigate the effect of GDM on neonatal SARS-CoV-2 infection, we divided the cohort into 3 groups according to the infectious status of SARS-CoV-2 and appearance of GDM, composed of the normal healthy pregnant women (Normal group), the SARS-CoV-2 infected pregnant women without GDM (SARS2 + noGDM group), and the infected women with GDM (SARS2 + GDM group), then we compared their differences in maternal, paternal, and fetal characteristics. As shown in **Table 2**, compared to the normal control group, the SARS2 + noGDM group had lower folic acid supplementation rate, age at menarche, and rate of neonatal SARS-CoV-2 infection. Consistently, the SARS2 + GDM group had higher age at pregnancy, pre-pregnancy BMI, vaccination for anti-SARS-CoV-2 rate and lower neonatal SARS-CoV-2 infection rate. In contrast, compared with the SARS2 + noGDM group, subjects in the SARS2 + GDM group had increased age at pregnancy and neonatal SARS-CoV-2 infection rate. For paternal factors, there were no statistically significant differences between groups.

**Table 2:** Description and comparison of observational variables between normal control and SARS-CoV-2 infected groups.

Variables	Normal (*N=518*)	SARS2+noGDM (*N=262*)	SARS2+GDM (*N=116*)	*p* overall
Maternal factors				
Age at pregnancy (yrs)	30.30 ± 4.02	30.62 ± 4.33	31.82 ± 3.87^*#^	0.002
Pre-pregnancy BMI (kg/m^2^)	26.31 ± 4.10	26.97 ± 3.76	27.55 ± 4.21^*^	0.004
Education background (n)				
Uneducated/Primary school graduate	23 (4.44%)	8 (3.05%)	4 (3.45%)	
High school graduate	43 (8.30%)	18 (6.87%)	6 (5.17%)	
University Graduate	380 (73.36%)	199 (75.95%)	92 (79.31%)	
Postgraduate	72 (13.90%)	37 (14.12%)	14 (12.07%)	
Vaccination for anti-COVID-19 (n)				0.010
No	130 (25.10%)	49 (18.70%)	16 (13.79%)	
Yes	388 (74.90%)	213 (81.30%)	100 (86.21%)*	
Folic acid supplementation (n)				0.004
No	20 (3.86%)	25 (9.54%)	10 (8.62%)	
Yes	498 (96.14%)	237 (90.46%)^*^	106 (91.38%)	
Medication (n) ^∫^				0.368
No	476 (91.89%)	233 (88.93%)	104 (89.66%)	
Yes	42 (8.11%)	29 (11.07%)	12 (10.34%)	
Age at first pregnancy (yrs)	27.44 ± 3.72	27.15 ± 3.39	27.80 ± 3.36	0.244
Gravidity (n)				0.553
0	207 (39.96%)	108 (41.22%)	41 (35.34%)	
≥1	311 (60.04%)	154 (58.78%)	75 (64.66%)	
Preterm birth (n)				0.079
0	512 (98.84%)	253 (96.56%)	114 (98.28%)	
≥1	6 (1.16%)	9 (3.44%)	2 (1.72%)	
Abortion (n)				0.057
0	489 (94.40%)	253 (96.56%)	105 (90.52%)	
≥1	29 (5.60%)	9 (3.44%)	11 (9.48%)	
Age at menarche (yrs)	14.08 ± 2.07	13.69 ± 0.83^*^	13.68 ± 0.85	0.003
Menstrual days (days)	5.83 ± 0.85	5.80 ± 0.80	5.77 ± 0.81	0.705
Menstrual Cycle regularity (n)				0.179
Regular	479 (92.47%)	251 (95.80%)	107 (92.24%)	
Irregular	39 (7.53%)	11 (4.20%)	9 (7.76%)	
Gestational age at delivery (wks)	39.02 ± 2.77	39.18 ± 1.30	39.09 ± 1.33	0.635
Delivery mode (n)				0.396
Vaginal delivery	313 (60.42%)	145 (55.34%)	68 (58.62%)	
Cesarean delivery	205 (39.58%)	117 (44.66%)	48 (41.38%)	
Conception method (n)				0.760
Artificial technology	23 (4.44%)	13 (4.96%)	7 (6.03%)	
Natural pregnancy	495 (95.56%)	249 (95.04%)	109 (93.97%)	
Paternal factors				
Age	30.81 ± 5.50	31.21 ± 5.66	31.88 ± 6.23	0.160
BMI	25.20 ± 3.43	25.33 ± 4.12	25.30 ± 4.11	0.890
Smoke (n)				0.725
No	370 (71.43%)	194 (74.05%)	85 (73.28%)	
Yes	148 (28.57%)	68 (25.95%)	31 (26.72%)	
Alcohol consumption (n)				0.993
No	329 (63.51%)	166 (63.36%)	73 (62.93%)	
Yes	189 (36.49%)	96 (36.64%)	43 (37.07%)	
Education background (n)				0.364
Uneducated/Primary school graduate	57 (11.00%)	25 (9.54%)	13 (11.21%)	
High school graduate	44 (8.49%)	22 (8.40%)	3 (2.59%)	
University Graduate	343 (66.22%)	184 (70.23%)	82 (70.69%)	
Postgraduate	74 (14.29%)	31 (11.83%)	18 (15.52%)	
Neonatal factors				
SARS-CoV-2 infection (n)				<0.001
Uninfected	273 (52.70%)	205 (78.24%)	75 (64.66%)	
Infected	245 (47.30%)	57 (21.76%)^*^	41 (35.34%)^*#^	
Sex of the newborn (n)				0.907
Male	261 (50.39%)	132 (50.38%)	61 (52.59%)	
Female	257 (49.61%)	130 (49.62%)	55 (47.41%)	

^*^compared with normal control, p<0.05.

^#^comparing with SARS-CoV-2 infection only group, *p*<0.05.

^∫^ any drugs taken during pregnancy.

SARS2, SARS-CoV-2 infection; GDM, gestational diabetes mellitus; BMI, body mass index.

### Risk factors for neonatal SARS-CoV-2 infection

**Figure 2:**
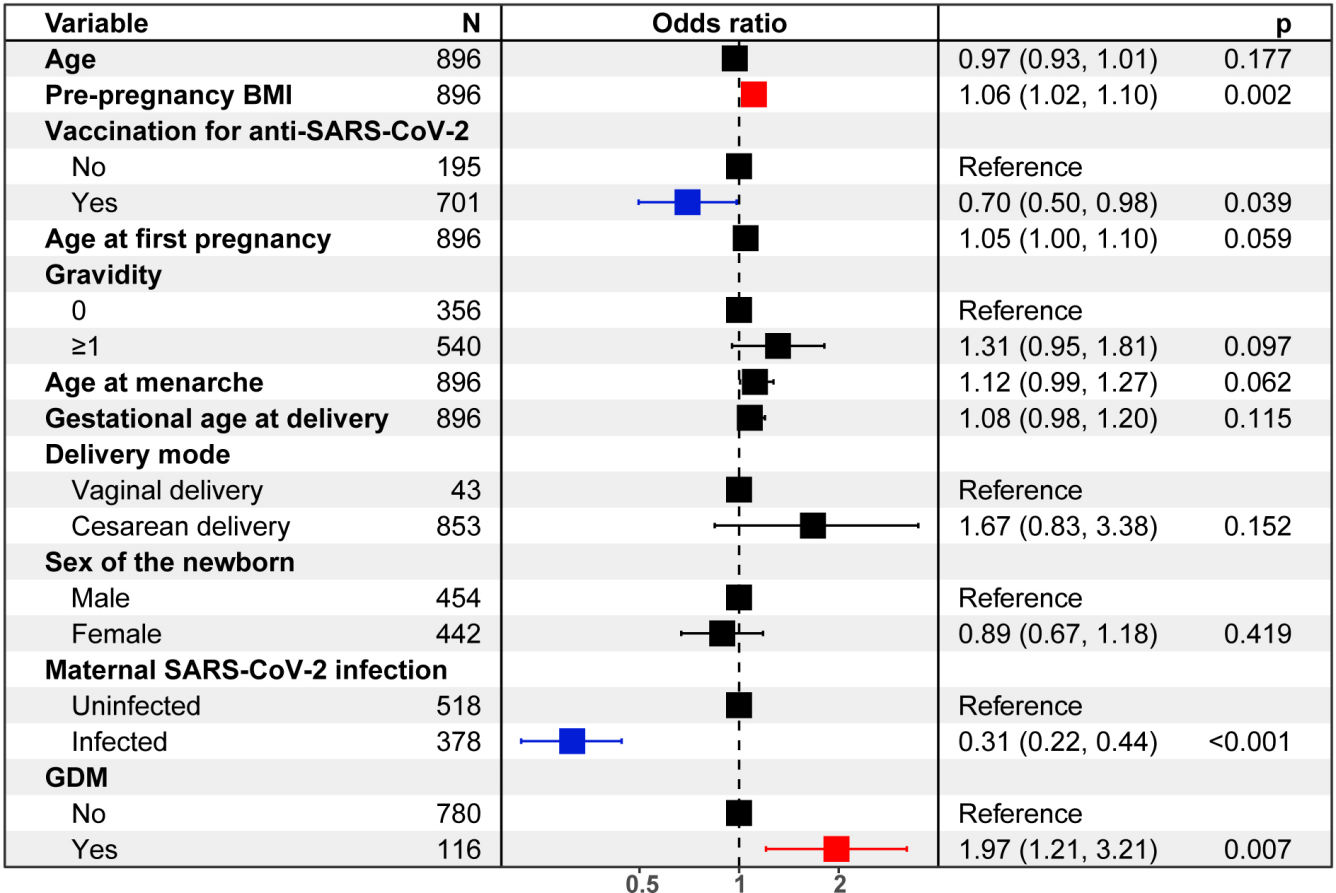
Logistic regression analysis quantifies risks of neonatal SARS-CoV-2 infection.

As shown in **Figure 2**, results of the logistic regression analysis showed that there was an increased risk of neonatal SARS-CoV-2 infection rate regarding the pre-pregnancy BMI (OR = 1.06, 95%CI: 1.02-1.10) and GDM (OR = 1.97, 95%CI: 1.21-3.21), while the decreased risk for neonatal infection was determined in anti-SARS-CoV-2 (OR = 0.70, 95%CI: 0.50-0.98), and maternal infection of SARS-CoV-2 (OR = 0.31, 95%CI: 0.22-0.44]).

## Discussion

This study aimed to investigate the associations between gestational diabetes mellitus (GDM), maternal SARS-CoV-2 infection, and neonatal SARS-CoV-2 infection. Our key findings suggest that coexistence of GDM and maternal SARS-CoV-2 infection was associated with higher odds of neonatal infection. Interestingly, maternal SARS-CoV-2 infection alone was associated with lower odds of neonatal infection, while higher pre-pregnancy BMI was associated with higher odds of neonatal infection. These findings contribute to the growing body of evidence on the complex interplay between maternal metabolic disorders, viral infections, and neonatal outcomes in the context of the COVID-19 pandemic. The observed effects of GDM and maternal SARS-CoV-2 infection on neonatal infection risk is particularly noteworthy and warrants further investigation.

Although the newly identified association adds new insights to the existing literature on GDM and neonatal outcomes, it’s important to note that previous studies have reported conflicting results regarding the impact of GDM on various neonatal outcomes during the pandemic. For instance, Norman et al. observed a higher risk of adverse neonatal outcomes, including increased likelihood of admission for neonatal care, neonatal morbidities of respiratory distress syndrome or any neonatal respiratory disorder, among infants born to mothers with SARS-CoV-2 (25). Kleinwechter et al. reported a higher incidence of stillbirth, death, and transfer to intensive care unit in neonates born to women with GDM during the COVID-19 pandemic (26). These contrasting findings highlight the complexity of the relationship between GDM and neonatal outcomes in the context of the COVID-19 pandemic. The discrepancies between our results and those of previous studies underscore the need for further research to elucidate the specific mechanisms and risk factors involved in neonatal SARS-CoV-2 infection and other adverse outcomes in pregnancies complicated by GDM.

The association between the GDM-SARS-CoV-2 coexistence and increased neonatal infection risk may be explained by several potential mechanisms. GDM is known to induce a state of chronic low-grade inflammation and alter placental function (27, 28). These changes, combined with the inflammatory response to SARS-CoV-2 infection, could potentially compromise the placental barrier and increase the risk of vertical transmission (29–31), supported by studies such as Faure‐Bardon et al. (32), who found evidence of SARS-CoV-2 proteins in placental tissues, suggesting potential vertical transmission. Additionally, GDM has been shown to affect the transfer of maternal antibodies to the fetus, which could impair the neonatal immune response to SARS-CoV-2 (21, 33), consistent with findings from Lampasona et al. (34), who reported reduced transplacental transfer of SARS-CoV-2 antibodies in pregnancies complicated by GDM.

Another crucial finding of this study was that maternal SARS-CoV-2 infection alone was associated with lower odds of neonatal infection, which seemed counterintuitive at first glance. It could potentially be explained by the transfer of maternal antibodies to the fetus, providing some level of passive immunity (21). This is supported by recent studies (35–37), which found that maternal SARS-CoV-2 infection during pregnancy was associated with higher levels of SARS-CoV-2-specific antibodies in cord blood, potentially conferring protection to the neonate. However, it’s important to note that other studies, such as Mullins et al., Giuliani at al. and Soheili et al. (38–40), have reported increased risks of adverse neonatal outcomes such as preterm delivery, low birthweight, neonate testing positive, neonatal mortality, and stillbirth associated with maternal SARS-CoV-2 infection. Specifically, Smith et al. pointed out in a meta-analysis that women with comorbidities such as preexisting diabetes mellitus and hypertension faced higher risk for COVID-19 severity and adverse pregnancy outcomes (fetal death, preterm birth, low birthweight) as compared to those free of comorbidities (41), highlighting the need for cautious interpretation of findings in this study.

The observed associations of higher pre-pregnancy BMI with increased neonatal infection risks align with previous studies, which suggest that obesity and multiple pregnancies may increase susceptibility to various infections (42, 43). Noteworthy, these factors may influence immune function and placental health, potentially affecting the risk of vertical transmission. For instance, several studies reported that pregnant women with higher BMI were at increased risk of severe COVID-19 outcomes, which could potentially impact neonatal health (44, 45). Similarly, Sahin et al. found that obesity was associated with increased risk of COVID-19 severity in pregnant women, although they did not specifically examine all neonatal outcomes (46).

Our observation of increased neonatal SARS-CoV-2 infection risk in pregnancies complicated by both GDM and maternal SARS-CoV-2 infection may be explained by the interplay of several factors. Firstly, the altered immune function associated with GDM could result in impaired host responses, potentially leading to higher maternal viral loads or prolonged infection, thus increasing the likelihood of vertical transmission or immediate postnatal infection (47). This effect may be compounded by the hyperinflammatory state induced by GDM, which, when combined with the acute inflammatory response to SARS-CoV-2, could compromise placental integrity, facilitating viral transmission across the placental barrier. Furthermore, GDM-related changes in placental structure and function, including abnormal uterine vascular remodeling, may create conditions more favorable for viral transmission (48). Finally, the potential reduction in maternal antibody transfer observed in GDM pregnancies could leave neonates with less passive immunity against SARS-CoV-2 (49), explaining their increased susceptibility to infection in the immediate postnatal period. These mechanisms likely interact in complex ways, collectively contributing to the increased risk we observed in the SARS2+GDM group compared to the SARS2+noGDM group.

While our study focused primarily on the risk factors for neonatal SARS-CoV-2 infection, it’s crucial to consider the potential consequences of such infections and why neonates may be particularly vulnerable. Neonatal SARS-CoV-2 infection can lead to a spectrum of outcomes (50), ranging from asymptomatic or mild disease to severe complications. Some neonates may experience respiratory distress, fever, poor feeding, lethargy, or gastrointestinal symptoms. In more severe cases, complications such as pneumonia, myocardial injury, or multisystem inflammatory syndrome in children (MIS-C) have been reported (51). The susceptibility of neonates to adverse outcomes can be attributed to several factors. First, their immature immune systems may struggle to mount an effective response against the virus, potentially leading to more severe or prolonged infections. Additionally, the rapid developmental changes occurring in various organ systems during the neonatal period may make them more vulnerable to the systemic effects of SARS-CoV-2. The potential for long-term developmental impacts, while still not fully understood, is also a concern, given the critical nature of this early life stage. Furthermore, neonates infected with SARS-CoV-2 may require separation from their mothers, potentially disrupting crucial bonding and breastfeeding, which could have additional health implications. Collectively, these factors underscore the importance of understanding and mitigating the risk of neonatal SARS-CoV-2 infection, particularly in high-risk groups such as those born to mothers with both GDM and SARS-CoV-2 infection.

Recent studies have highlighted the potential impact of fertility procedures on the immune status of children. For instance, Huniadi et al. explored fertility predictors in intrauterine insemination (IUI) and emphasized the importance of factors such as endometrial thickness, sperm concentration, and motility in determining the success of IUI (52). These factors not only influence the likelihood of pregnancy but may also affect the intrauterine environment, potentially impacting fetal immune development. Similarly, Zaha et al. investigated the use of autologous platelet-rich plasma (PRP) in infertility treatments, comparing infusion versus injectable PRP. They found that PRP, particularly when injected sub-endometrially, improved endometrial thickness and pregnancy rates in IVF patients, which suggests that PRP may enhance endometrial receptivity, which could have downstream effects on the immune environment of the developing fetus (53). While our study did not specifically examine the role of ART in neonatal SARS-CoV-2 infection, it is plausible that fertility treatments could modulate maternal immune responses and placental function, thereby influencing neonatal susceptibility to infections. For example, the altered immune milieu associated with ART might affect the transfer of maternal antibodies to the fetus, a critical factor in neonatal immunity. Future research should explore whether children conceived through ART, particularly those involving PRP or other immune-modulating treatments, exhibit differences in immune responses to infections such as SARS-CoV-2. This could provide valuable insights into the long-term health outcomes of children born through these procedures, especially in the context of emerging infectious diseases.

For future studies, which should focus on elucidating the mechanisms underlying the interaction between GDM and SARS-CoV-2 infection, as well as its impact on neonatal outcomes. By involving more detailed examination of placental pathology, as suggested by Boyraz et al. (54), we may distinct specific placental pathology patterns in SARS-CoV-2-infected pregnancies. Additionally, research into potential interventions to mitigate the increased risk associated with this interaction is warranted. For instance, studies could explore whether tighter glycemic control in GDM patients with SARS-CoV-2 infection could reduce the risk of neonatal infection, building on work by Bhatti et al. who found that poor glycemic control was associated with worse COVID-19 outcomes in diabetic patients (55).

Nonetheless, this study has several limitations that should be considered. As an observational study, we cannot establish causal relationships between the variables. The retrospective nature of the study may introduce recall bias, and we cannot rule out the influence of unmeasured confounding factors. Our study was conducted in a single center, which may limit its generalizability to other populations, which is particularly important given that COVID-19 outcomes have been shown to vary across different geographic regions and healthcare systems. For future prospective, multi-center studies with larger sample sizes could help validate our findings and provide more robust evidence. Moreover, our study did not account for potential variations in SARS-CoV-2 strains, which could influence transmission and outcome patterns. Recent study by Whitaker et al. has highlighted the importance of considering variant-specific effects in COVID-19 research (56). Additionally, we did not have data on the timing of SARS-CoV-2 infection during pregnancy, which could potentially influence neonatal outcomes, as suggested by Movahedi et al. and Sahin et al. who found that third-trimester infections were associated with higher rates of certain complications (57, 58).

## Conclusion

To sum up, our study provides important insights into the complex relationships between GDM, maternal SARS-CoV-2 infection, and neonatal outcomes. The observed coexistence of GDM and maternal SARS-CoV-2 infection on neonatal infection risk underscores the importance of comprehensive care for pregnant women during the COVID-19 pandemic, particularly those with metabolic disorders. Our findings both support and contrast with various aspects of previous research, highlighting the complexity of these relationships and the need for continued investigation. Further research is needed to fully understand the mechanisms underlying these associations and to develop targeted interventions to improve maternal and neonatal outcomes in the context of the ongoing pandemic.

## Data Availability

The original contributions presented in the study are included in the article/supplementary material. Further inquiries can be directed to the corresponding author.
